# Prevalence of osteoporosis in patients awaiting total hip arthroplasty

**DOI:** 10.1590/1413-78522015230100981

**Published:** 2015

**Authors:** Vitor Rodrigues Domingues, Gustavo Constantino de Campos, Pérola Grimberg Plapler, Márcia Uchôa de Rezende

**Affiliations:** Universidade de São Paulo, Faculdade de Medicina, Hospital das Clinicas, São Paulo, Brazil, Instituto de Ortopedia e Traumatologia do Hospital das Clinicas da Faculdade de Medicina da Universidade de São Paulo (FMUSP), São Paulo, Brazil

**Keywords:** Osteoarthritis, Osteoporosis, Bone density, Arthroplasty, replacement, hip

## Abstract

**Objective:**

To evaluate the prevalence of osteoporosis in patients awaiting total hip arthroplasty.

**Method:**

Twenty-nine patients diagnosed with hip osteoarthritis awaiting primary total arthroplasty of the hip answered WOMAC questionnaire, VAS and questions about habits, osteoporosis and related diseases. Bone mineral densitometry of the lumbar spine and hips and laboratory tests (complete blood count and examination of calcium metabolism) were performed. Weight and height were measured to calculate body mass index (BMI). The evaluated quantitative characteristics were compared between patients with and without osteoporosis using the Mann-Whitney tests.

**Results:**

Thirteen men and 16 women with a mean age of 61.5 years old, WOMAC 51.4; EVA 6.4 and BMI 27.6 were evaluated. The prevalence of osteoporosis was 20.7%, and 37.9% had osteopenia. Patients with osteoporosis were older than patients without osteoporosis (p=0.006). The mean bone mineral density of the femoral neck without hip osteoarthritis was lower than the affected side (p=0.007). Thirty-five percent of patients did not know what osteoporosis is. Of these, 30% had osteopenia or osteoporosis.

**Conclusion:**

osteoarthritis and osteoporosis may coexist and the population waiting for total hip arthroplasty should be considered at risk for the presence of osteoporosis. Level of Evidence III, Observational Study.

## INTRODUCTION

Osteoarthritis (OA) and osteoporosis are two age-related conditions, both common in post-menopause women.^1^^,^ 2 The relationship between the two diseases remains unclear, even after more than 40 years since the first description of an apparent inverse relationship.^3^ According to some authors the incidence of osteoporosis is inversely correlated with the incidence of OA,^4.5^, i.e., the presence of OA would protect against osteoporosis. Recent evidence, however, point to a bone loss in patients with OA,^6^^-^ 8 even in the early stages of disease.^9^

Total hip arthroplasty (THA) is one of the most successful surgical procedures in orthopedics, considered by some as the "surgery of the century".^10^ More than one million arthroplasties are performed annually worldwide, and projections indicate that this number will double in the next decade.^11^

Cementless techniques are increasingly used, but have never been systematically evaluated for its use in osteoporotic patients. It is known that poor bone quality may affect the initial stability of non-cemented implants.^12^

Osteoporosis can lead to four possible complications of cementless THA: increased migration (subsidence) of the rod, delay in osteointegration, increased risk of periprosthetic fracture and risk of late failure.^2^ The presence of osteoporosis in patients with OA of the hip has, therefore, important implications both in disease progression, as from a surgical point of view, due to the potential of adversely influence the result of THA.^6^ Thus, patients at risk for developing osteoporosis should be investigated. This study aims to assess the prevalence of osteoporosis in patients awaiting total hip arthroplasty.

## METHODS

This study was performed at the Department of Orthopedics and Traumatology, *Instituto de Ortopedia e Traumatologia da Faculdade de Medicina da Universidade de São Paulo* (DOT FMUSP). It has been approved by the Ethics Committee for Research Project Analysis (CAPPesq) under n° 0338/10.

The first 50 patients on the waiting list to perform primary total hip arthroplasty at *Instituto de Orthopedia e Traumatologia* do Hospital das Clínicas da FMUSP were invited to participate in the study. Inclusion criteria were: hip OA confirmed by radiography, to be participating in the primary total hip arthroplasty program, understanding and agreeing to the informed consent form. Exclusion criteria were history of Paget's disease or Osteogenesis imperfecta, rheumatic diseases, presence of any implant on the hip under study, contralateral hip or spine.

The free and informed consent, as well as a questionnaire about previous fractures, menopause, smoking, alcohol use, estrogen, calcium and vitamin D supplementation were applied by the researcher. The use of any drug for osteoporosis was also questioned. Weight and height were measured to calculate body mass index (BMI). Patients also completed the Western Ontario questionnaire and the McMaster Universities Osteoarthritis Index (WOMAC)^13^ and the Visual Analogue Scale for pain (VAS).^14^

Bone mineral density (BMD) was measured by bone densitometry of the lumbar spine and bilateral proximal femur performed by a DEXA machine (Dual Energy X-Ray Absorptiometry) Lunar DPX. Blood samples were collected and laboratory tests performed for detection of bone metabolic pathologies and assess prevalence of vitamin D insufficiency. Blood tests included: complete blood count, serum calcium, calcium in 24 hours urine, phosphorus, parathyroid hormone, 25(OH)VitD, alkaline phosphatase and osteocalcin (bone metabolism markers).

Radiographs in AP+P load of the affected hip were evaluated and a radiological classification was performed by two independent observers using the Kellgren-Lawrence classification.^15^

### Statistical Analysis

Statistical analysis was performed using Excel 2003 software (Microsoft Corporation, Redmond - WA, USA) and SPSS 20.0 (IBM, New York - NY, USA). The tests were done at 5% significance level.

The qualitative characteristics evaluated with absolute and relative frequencies and quantitative measures using summary measurements were first described (mean, standard deviation, median, minimum and maximum).

The diagnosis of osteoporosis was defined in patients with bone mineral density values less than 2.5 standard deviation peak values in young adults (T <-2.5 score) at any location. Osteopenia was defined in patients with bone mineral density values between -1 and -2.5 standard deviation peak values in young adults at any location, as defined by the World Health Organization (WHO). The prevalence of osteoporosis was described according to each qualitative characteristic assessed and checked for association with the use of Fisher's exact test or likelihood ratio test when the characteristics has more than two categories. The assessed quantitative characteristics were described according to the presence of osteoporosis and compared between patients with and without osteoporosis using Mann-Whitney tests.

Bone mineral density at the femoral neck and total femur were described in the hip side with and without osteoarthritis and sides were compared by generalized estimating equations with symmetric component correlation matrix between the sides assuming normal distribution of BMD as a function of identity link.

## RESULTS

Of the 50 patients enrolled, 29 performed all the exams and were included in the final statistical analysis. Table 1 shows the characteristics of the study population. The prevalence of osteoporosis in this patient was 20.7% (six patients) and of osteopenia was 37.9% (11 patients).

**Table 1.** Description of qualitative characteristics assessed and confidence interval for prevalence of osteoporosis in patients with hip arthrosis.

Variable	Description (N=29)
Gender	
Masculine	13 (44.8)
Feminine	16 (55.2)
Ethnic group	
White	24 (82.8)
Non white	5 (17.2)
Hip	
Right	9 (31)
Left	14 (48.3)
Bilateral	6 (20.7)
Smoker	
No	18 (62.1)
Yes	11 (37.9)
Alcohol user	
No	26 (89.7)
Yes	3 (10.3)
Menopause*	
No	6 (35.3)
Yes	11 (64.7)
Hormone Replacement Therapy*	
No	13 (76.5)
Yes	4 (23.5)
vitamin D	
No	24 (82.8)
Yes	5 (17.2)
calcium	
No	21 (72.4)
Yes	8 (27.6)
alendronate	
No	25 (86.2)
Yes	4 (13.8)
risedronate	
No	29 (100)
zoledronic acid	
No	29 (100)
Diabetes	
No	26 (89.7)
Yes	3 (10.3)
Fracture on studied hip	
No	23 (79.3)
Yes	6 (20.7)
Fracture on contralateral hip	
No	26 (89.7)
Yes	3 (10.3)
Spine fracture	
No	28 (96.6)
Yes	1 (3.4)
Knows what osteoporosis is	
No	10 (34.5)
Yes	19 (65.5)
Osteoporosis	
No	23 (79.3)
Yes	6 (20.7)
CI (95%)	(6.0 - 35.4)

* Somente 17 mulheres.

The prevalence of inadequate serum levels of vitamin D was also high. Only 16.6% of patients had levels considered normal (>30 ng/ml). The prevalence of vitamin D insufficiency (serum level between 20 and 30 ng/ml) was 45.8% and the prevalence of vitamin D deficiency (<20 ng/ml) was 37.5%. Thirty-five percent of patients did not know what osteoporosis was.

Table 2 shows that the mean age of the patients was 61.5 years old (St. Dev 15.2 years old), with mean VAS higher than 5 and WOMAC scale above the mean scale value (51.4 points).

**Table 2.** Description of quantitative characteristics assessed in patients.

Variables	Mean	St. Dev.	Median	Minimum	Maximum	N
Age (years old)	61.5	15.2	64	23	83	29
BMI (Kg/m2)	27.6	3.8	27.58	20.3	35.4	29
WOMAC	51.4	20.8	54	8	88	27
VAS	6.4	3.1	7	1	10	27
Ca2+ (mg/dl)	9.2	1.0	9.3	4.8	10.3	29
P (mg/dl)	3.4	0.5	3.3	2.2	4.6	29
AP (U/l)	85.9	40.3	77	4	222	29
PTH (pg/ml)	68.9	42.9	52	18	199	23
Vit. D (ng/ml)	22.2	7.8	21	9	41	24

Table 3 shows that there was no statistically significant association between the presence of osteoporosis with the qualitative characteristics of the patients (p>0.05).

**Table 3.** Description of osteoporosis prevalence osteoporosis according to qualitative characteristics of patients and results of association tests.

Variables	Osteoporosis				Total	p
	No		Yes			
	n	%	n	%		
Gender						0.663
Masculine	11	84.6	2	15.4	13	
Feminine	12	75.0	4	25.0	16	
Ethnic group						0.265
White	20	83.3	4	16.7	24	
Non white	3	60.0	2	40.0	5	
Hip						0.101#
Right	5	55.6	4	44.4	9	
Left	13	92.9	1	7.1	14	
Bilateral	5	83.3	1	16.7	6	
Smoker						0.164
No	16	88.9	2	11.1	18	
Yes	7	63.6	4	36.4	11	
Alcohol user						>0.999
No	20	76.9	6	23.1	26	
Yes	3	100.0	0	0.0	3	
Menopause						>0.999
No	5	83.3	1	16.7	6	
Yes	8	72.7	3	27.3	11	
Hormone Replacement Therapy						0.219
No	11	84.6	2	15.4	13	
Yes	2	50.0	2	50.0	4	
vitamin D						0.553
No	18	75.0	6	25.0	24	
Yes	5	100.0	0	0.0	5	
calcium						0.148
No	15	71.4	6	28.6	21	
Yes	8	100.0	0	0.0	8	
alendronate						>0.999
No	20	80.0	5	20.0	25	
Yes	3	75.0	1	25.0	4	
Diabetes						0.100
No	22	84.6	4	15.4	26	
Yes	1	33.3	2	66.7	3	
Fracture on studied hip						>0.999
No	18	78.3	5	21.7	23	
Yes	5	83.3	1	16.7	6	
Fracture on contralateral hip						0.515
No	21	80.8	5	19.2	26	
Yes	2	66.7	1	33.3	3	
Spine fracture						>0.999
No	22	78.6	6	21.4	28	
Yes	1	100.0	0	0.0	1	
Knows what osteoporosis is						0.633
No	9	90.0	1	10.0	10	
Yes	4	73.7	5	26.3	19	

Result of Fisher's exact test; # Result of likelihood ratio test.

Table 4 shows that patients with hip osteoarthritis who had osteoporosis were statistically significant older than patients without osteoporosis (p=0.006), the other parameters were similar between patients with and without osteoporosis (p>0, 05).

**Table 4.** Description of quantitative characteristics assessed regarding presence of osteoporosis and results of comparative tests.

Variable	Osteoporosis	Mean	St. Dev.	Median	Minimum	Maximum	N	p
Age (years old)	No	58.09	14.68	59	23	79	23	0.006
	Yes	74.50	9.48	77	56	83	6	
BMI (kg/m^2^)	No	27.44	3.70	27.56	20.3	33.7	23	0.581
	Yes	28.05	4.42	28.35	22.9	35.4	6	
WOMAC	No	50.90	19.51	54	8	88	21	0.476
	Yes	53.00	26.9	62.5	19	84	6	
VAS	No	6.19	3.19	7	1	10	21	0.441
	Yes	7.33	2.81	8	2	10	6	
Ca^2^+ (mg/dl)	No	9.12	1.04	9.3	4.8	10.1	23	0.477
	Yes	9.52	0.58	9.35	8.8	10.3	6	
P (mg/dl)	No	3.32	0.50	3.3	2.2	4.6	23	0.414
	Yes	3.53	0.46	3.35	3	4.1	6	
AP (U/l)	No	86.30	38.77	74	42	222	23	0.773
	Yes	84.52	49.94	90	4	153	6	
PTH (pg/ml)	No	71.89	46.41	62	18	199	19	0.667
	Yes	54.75	16.62	48.5	43	79	4	
vit D (ng/ml)	No	23.00	8.22	24	9	41	20	0.241
	Yes	18.25	3.10	19	14	21	4	

Result of Mann-Whitney test.

Table 5 shows that mean BMD of the femur neck of the hip without osteoarthritis side was lower than side with osteoarthritis (p=0.007). 

**Table 5.** Description of BMD of the femur neck and total femur according to the side of affected hip and results of comparison between sides.

Variable	Side of the hip	Mean	St. Dev.	Median	Minimum	Maximum	N	p
Femur neck	Arthrosis	-1.03	1.19	-1.2	-2.8	2.4	20	0.007
(T-score)	No arthrosis	0.60	2.92	0	-3.3	9.1	28	
Total femur	Arthrosis	-1.13	1.24	-1.55	-3	2	20	0.148
(T-score)	No arthrosis	-0.53	1.77	-0.8	-4	3	28	

Result of Wald test.

## DISCUSSION

The number of arthroplasties performed annually is increasing,^11^ and tends to increase, since our population is growing older.^16^ Osteoarthritis patients who require joint replacement are predominantly elderly women, a population at high risk for developing osteoporosis. This is the first study to evaluate the relationship between osteoporosis and osteoarthritis in the Brazilian population.

The main finding of this work are in accordance with the world literature, showing a prevalence of osteoporosis of 20.7% in patients awaiting THA, which is compatible to the prevalence found in the general population.^17^ These findings support the conclusion that osteoporosis and osteoarthritis can be found in the same patient, confirming recent data from the literature.

Our results are similar to those found by Glowacki *et al.*,^1^ who performed similar preoperative analysis of 68 postmenopausal women scheduled for undergoing THA, which found hidden osteoporosis in 25% of patients. Makinen *et al.* 7 found a prevalence of 28% of osteoporosis and osteopenia in 45% of patients scheduled for THA, besides a 36% prevalence of vitamin D deficiency. These two studies evaluated a population formed only by women, which explains the higher prevalence of osteopenia and osteoporosis they found. Our study, however, included 44% of men who, in general, have lower prevalence of osteoporosis.

Another important finding was the difference between the values of bone mineral density of the osteoarthritic hip and contralateral hip, with higher densities in the sick hip. This can be explained by the presence of bone sclerosis in the affected joint. This finding may also explain the lower prevalence of femoral neck fractures in patients with hip osteoarthritis.^18^ Lingard *et al.* 6 found a 23% prevalence of osteoporosis among 199 patients scheduled for total hip arthroplasty. Low bone density was detected most commonly in the forearm (14%) in the lumbar spine (8.5%) and proximal femur (8.2%). It is important, therefore, that the bone densitometry in osteoarthritic patients always include other non-affected anatomical regions, so not to over-estimate the bone mineral density in these patients.

The present study has limitations. First, we evaluated 29 patients, only 58% of those initially recruited. The low participation of patients may be related to their difficulty to go to the hospital due to pain or lower functional capacity, data that may alter EVA and WOMAC analysis. Although not representing all patients studied, we believe that the sample represents the group of patients to be studied. Second, we could benefit from a control group of individuals without arthritis matched by age and gender. The strengths of our study are the homogeneity of the population studied, all in the final stage, the assessment of osteoporosis by densitometry, which eliminates self-reported errors on surveys, conducting densitometry study in various anatomic sites, besides performing other blood-related bone metabolism tests.

In the present study, the majority of patients (58.6%) had osteopenia or osteoporosis. A considerable number of patients (35%) did not know what osteoporosis is, nor did they know they had such a disease. Of these, 30% had osteopenia or osteoporosis, which points out the inattention to the condition.

## CONCLUSION

Contrary to general belief, osteoarthritis seems not to protect against osteoporosis, and people awaiting total hip arthroplasty should be considered at risk for the presence of osteoporosis.
